# The Role of Exercise on Fatigue Among Patients With Multiple Sclerosis in the King Fahad Hospital, Madinah, Saudi Arabia: An Analytical Cross-Sectional Study

**DOI:** 10.7759/cureus.42061

**Published:** 2023-07-18

**Authors:** Zahrah I Alsharif, Farah A Mansuri, Yasser A Alamri, Nouf A Alkalbi, Maha M Almutairi, Ahmed F Abu Alkhair

**Affiliations:** 1 Department of Preventive Medicine, Saudi Board of Preventive Medicine, Ministry of Health, Madinah, SAU; 2 Department of Family and Community Medicine, Taibah University, Madinah, SAU; 3 Department of Neurology, King Salman Bin Abdulaziz Medical City, Madinah, SAU

**Keywords:** patients with multiple sclerosis, ksa, exercises, fatigue, multiple sclerosis

## Abstract

Background

Multiple sclerosis (MS) is a chronic autoimmune disease caused by multiple factors. It can lead to many physical and mental symptoms. Fatigue is one of the most commonly mentioned complaints among MS patients that can affect their quality of life. Physical activity has many benefits for the physical and mental health of patients with MS.

Aim

To assess the role of exercise on fatigue among patients with multiple sclerosis and identify the relationship between depression, sleep quality, sociodemographic variables, and fatigue.

Methods

This is an analytical cross-sectional study based on a sample size of 235 patients recruited from the MS clinic at King Fahad Hospital (KFH) in Madinah. The outcome of the study was fatigue among MS patients. Data were collected through telephone calls from February to May 2022 using a structured questionnaire and scales, such as the Godin Leisure-Time Exercise Questionnaire (GLTEQ), Modified Fatigue Impact Scale (MFIS), Patient Health Questionnaire (PHQ2), and Pittsburgh Sleep Quality Index (PSQI). Data were analyzed through SPSS version 20 (IBM Corp., Armonk, NY, USA). The correlation coefficient (r), Chi-square tests, and simple and multiple logistic regression were used as found appropriate.

Results

Out of the total samples, 37.4% were male and 62.6% were female. The median age of patients was 36 years. The prevalence of fatigue was 37% among patients, with a reported median fatigue score of 26. It was found that 63% of the patients were physically inactive; 32.2% were overweight, 14.2% were obese; 63.8% of patients had poor sleep quality. The fatigue score was negatively correlated with the GLTEQ score, but the results were not significant (r=−0.066; P-value (level of significance)=0.335). Nonetheless, a moderately significant correlation was observed between the MFIS and PSQI and MFIS and PHQ2 (r=0.505, P=<0.001 and r=0.520, P=<0.001, respectively). The Chi-square test showed a significant association between fatigue and progressive types of MS, the primary progressive MS (PPMS), secondary progressive MS (SPMS), and relapsing-remitting MS (RRMS) (odds ratio (OR)=4.4; 95% confidence interval (CI): 2.1-8.9), P=<0.001). Depressed patients were 9.7 times more likely to develop fatigue compared to non-depressed patients (P=<0.001). Those with poor sleep quality were 4.6 times more likely to develop fatigue compared to those with good sleep quality (P=<0.001). Fifty-six percent of fatigue among MS patients were predicted by low income, progressive types, unemployment, obesity, depression, and poor sleep quality.

Conclusion

Fatigue is a major complaint among MS patients. Most of the patients were found to be physically inactive, depressed, and have poor sleep quality. This study found an association between physical inactivity and fatigue, but the results were not significant. There was a significant association between sociodemographic factors like low income and unemployment, poor sleep quality, obesity, progressive types of MS, depression, and fatigue. Encouraging exercise practice and implementing a regular exercise program are needed, along with weight management plans. Further studies and psychological support meetings are required, with the importance of a holistic approach to patient care.

## Introduction

Multiple sclerosis (MS) is a chronic autoimmune disease in which the axons in the central nervous system are demyelinated. Many factors may contribute to the development of the disease, including environmental and genetic factors, yet the main cause remains unknown [1]. Symptoms of the disease include numbness, weakness, fatigue, imbalance, low mood, depression, and visual and bladder problems [2]. According to a meta-analysis conducted on Iranian patients, the most common MS symptoms are fatigue and motor dysfunction, with an estimated prevalence of 71.1% and 56.3%, respectively [3]. The most common MS symptoms, according to the national MS registry in the Kingdom of Saudi Arabia (KSA), included the following: of the patients across all regions, muscle weakness was most common (57.1%), followed by visual symptoms (48.2%), and sensory symptoms (47.3%). The prevalence of fatigue in Saudi Arabia was found to be higher in females compared to males (61.95/100,000 among Saudi nationals) [4].

Fatigue was rarely listed as a symptom of MS until Freal published a report in which 78% (n=656) of patients with MS mentioned fatigue as one of their symptoms. Of the total patients, 28% mentioned that fatigue made symptoms more apparent, 43% mentioned that fatigue was similar to MS exacerbation, and 11% mentioned that it was the same as exacerbation. Due to these difficulties in distinguishing between true relapse symptoms and fatigue, under-reporting of relapses might occur [5]. In the previous 10-15 years, many researchers have found that engaging in physical activity helps patients with MS to manage symptoms, restore function, and optimize their quality of life (QOL). Exercise training was also found to be associated with improvements in muscular and cardiorespiratory fitness among patients with MS [6,7].

Hadjimichael et al. studied fatigue in 18,595 North American Research Committee on Multiple Sclerosis (NARCOMS) registrants in November 2002 [8]. In that study, the researchers used the Modified Fatigue Impact Scale (MFIS) and Fatigue Severity Scale (FSS), as well as questions related to symptom management. The progressive relapsing MS (PRMS) type had a higher prevalence of severe fatigue compared to relapsing-remitting MS (RRMS) and primary progressive MS (PPMS). Patients with severe fatigue had higher MFIS scores. Fatigue scores in both FSS and MFIS were significantly increased when they were examined relative to the duration of MS for about the first 14 years.

Razazian et al. carried out a systematic review and meta-analysis to measure the impact of physical activities on fatigue among patients with MS. The study included 31 clinical trials and used the random effect to obtain the outcome. The standardized estimated mean difference in the fatigue score using the FSS score between groups before and after the intervention was 23.8 ± 6.2 and 16.9 ± 3.2, respectively. Based on this result, the study concluded that physical activities improve physical fatigue in patients with MS [9].

Halawani et al. conducted a case-control study to study the lifestyle factors, environmental factors, and socioeconomic factors that can lead to the development of MS in the western region of Saudi Arabia. It was found that obesity, smoking, and measles infections increase the risk of getting MS. On the other hand, it was found that vigorous exercise is not associated with the development of MS [10].

The primary objective of our study: to find the association between exercise and fatigue among MS patients in KFH, Madinah. Secondary objectives: to find the association between sociodemographic variables, depression, sleep quality, and fatigue among MS patients.

The prevalence of MS has been found to be increasing in KSA. It becomes a huge burden on patients and creates a huge economic burden for society by affecting young adults in their most productive years.

The study aims to assess the role of exercise on fatigue among patients with multiple sclerosis and identify the relationship between depression, sleep quality, sociodemographic variables, and fatigue.

## Materials and methods

For this analytical cross-sectional study, participants were selected from the MS clinic at the King Fahad Hospital (KFH) in Madinah between February and May 2022. The sample size was calculated to be 235 using the OpenEpi software program (population size: 600, expected frequency: 50%, margin of error: 5% and 95% CI). The whole list of patients was contacted until 211 samples were obtained (89% response rate). The inclusion criterion was the presence of MS in patients from the MS clinic at KFH, according to McDonald's criteria. The exclusion criteria were severe psychiatric disorders, other comorbidities that interfered with physical activities (for example, musculoskeletal problems), the terminal illness of MS, and pregnancy. Fatigue was the dependent variable, while sociodemographic factors, smoking, sleeping habits, exercise, use of vitamin D, body mass index (BMI), type of MS, number of relapses per year, use of multivitamins, use of MS medications, other comorbidities, family history of MS, duration of illness, and depression were independent variables.

Ethical approval was obtained from the Institutional Review Board (IRB), General Directorate of Health Affairs in Madinah (approval number: 001-2022). Informed consent was also obtained from the participating patients. Confidentiality and privacy of the data were ensured. The patient had the right to participate voluntarily and could withdraw at any time from the study.

Data were collected by senior neurology residents from patients via a telephone call and using a questionnaire, which included questions about sociodemographic status, type of MS, duration of illness, number of relapses per year, use of MS medications, use of vitamin D, use of multivitamins, family history of MS, exercise, sleeping habits, and depression. Regarding the (living) variable, patients were asked if they live in the city or in one of the villages near Madinah. In addition to using the Godin Leisure-Time Exercise Questionnaire (GLTEQ), the MFIS to measure fatigue, the Patient Health Questionnaire (PHQ2) to measure depression in patients, and the Pittsburgh Sleep Quality Index (PSQI) was used.

Measures

MFIS is a reliable and valid tool to measure fatigue. It measures the physical, cognitive, and psychosocial aspects of fatigue. The reported internal consistency of the MFIS scores was excellent according to the subsequent Cronbach's alpha values: total, 0.81; physical, 0.91; psychosocial, 0.81; and cognitive, 0.95 [11]. PHQ2 was used as a screening tool for major depressive disorder (MDD), with a pooled sensitivity of 0.76 and specificity of 0.87, with a cutoff point of ≥3. In addition to this, the pooled sensitivity was 0.91 and the specificity was 0.70, with a cutoff point of ≥2 [12]. PSQI was also added to assess sleep problems in patients with MS. In previous studies, Cronbach’s alpha was 0.77, demonstrating the acceptable reliability of the questionnaire [13]. GLTEQ is a valid and simple tool used previously in patients with MS to assess physical activity [14].

Scoring

For MFIS, the cutoff point was a score of ≥38, and the score was directly proportional to the effect of fatigue on the life of the patient [11]. PSQI is a global score of sleep quality calculated by summation of the component scores and ranges from 0 to 21 with better quality of sleep associated with lower scores [13]. Sleep quality was determined to be: 0-4 indicating good sleep quality, and 5-21 indicating poor sleep quality. For PHQ2, the cutoff score was 2 [12]. The cutoff point for the "GLTEQ" scale was 24. Patients who scored 24 units or more were considered active, and patients with fewer scores were considered inactive [15].

Statistical analysis

Data were analyzed using SPSS version 20 (IBM Corp., Armonk, NY, USA). Continuous variables were analyzed as mean and standard deviation (SD) or median with interquartile range (IQR) in the case of non-normally distributed data. Categorical data were analyzed in terms of frequency and proportion. The correlation coefficient (r) test was used to find the relationship between the PHQ2 score, GLTEQ score, PSQI score, and MFIS; the Chi-squared test was used to find an association between categorical independent variables and the outcome fatigue; and simple logistic regression was used to find an association between independent variables with more than two categories and fatigue; and a multiple logistic regression model was used for all significant variables. The significance level was predetermined at a P-value of <0.05 for all tests. An external pilot study was conducted on individuals, and information was not included in the analysis.

## Results

The response rate was 89% (211 of 235 participants). The data were not normally distributed. Skewness was found in the data, and Kolmogorov-Smirnov and Shapiro-Wilk tests were significant for all variables.

Table 1 shows the main characteristics of the participants by frequency and percentage. Among responders, 37.4% were male (n=79) and 62.6% were female (n=132). The median age of patients was 36 years. BMI was calculated with a median of 24.50 and an IQR of 6.3. Of the total patients, 32.2% were overweight (n=68) and 14.2% were obese (n=30). Among the participants, 63.5% were married (n=134) and 36.5% were unmarried (n=77). The median number of children was 1 with an IQR of 4. According to the smoking data, 26.1% were smokers (n=55) and 73.9% were non-smokers (n=156) (Table 1).

**Table 1:** Characteristics of patients with MS (n=211). F: frequency; BMI: body mass index; MS: multiple sclerosis.

Variable	F (%)
Age	
Young adults	107 (50.7)
Middle-aged	96 (45.5)
Elderly	8 (3.8)
BMI	
Underweight	12 (5.7)
Normal	101 (47.9)
Overweight	68 (32.2)
Obese	30 (14.2)
Gender	
Male	79 (37.4)
Female	132 (62.6)
Employment status	
Student	16 (7.6)
Employed	94 (44.5)
Unemployed	101 (47.9)
Educational level	
School	79 (37.4)
University	125 (59.2)
Postgraduate studies	7 (3.3)
Marital status	
Married	134 (63.5)
Unmarried	77 (36.5)
Income (Saudi Riyal)	
Less than 4000	26 (12.3)
4000-7999	35 (16.6)
8000-11,999	30 (14.2)
12,000-15,999	35 (16.6)
16,000-19,999	46 (21.8)
More than 20,000	39 (18.5)
Living	
In a city	200 (94.8)
In a village	11 (5.2)
Type of house	
Own house	128 (60.7)
Rental house	83 (39.3)
Smoking	
Yes	55 (26.1)
No	156 (73.9)
Family history of MS	
Yes	44 (20.9)
No	167 (79.1)
Sleeping hour	
Before midnight	59 (28)
After midnight	152 (72)
Sleep quality	
Poor sleep quality	134 (63.5)
Good sleep quality	77 (36.5)

Table 2 shows the clinical characteristics of patients with MS. According to the types of MS, 79.6% of patients had RRMS, 15.2% had PPMS, and 5.2% had secondary progressive MS (SPMS). The median time since diagnosis was 48 months, and the median number of relapses per year was 1 with an IQR of 2. Of the total patients, 74.4% were on disease-modifying therapy (DMT) (n=157) and 25.6% were not (n=54). Sixty-three patients out of 211 recorded their vitamin D level, with the mean value being 35.51. It was found that 68.2% of patients (n=144) were taking vitamin D supplements.

About the scales used in this study, the median fatigue score was 26 (IQR, 37) with a cutoff on the MFIS of 38; 37% were categorized as fatigued (n=78). The median PSQI score of the sample was 5 (IQR, 3.50).

The median score on the GLTEQ was 9 with an IQR of 32. Of the total patients, 37% were considered physically active (n=78), and 63% were physically inactive (n=133). Regarding PHQ2, the median score was 2 with an IQR of 3, with 54% of patients being categorized as depressed (n=144) and 46% as non-depressed (n=97). The correlation between MFIS, PSQI, PHQ2, and GLTEQ was studied using Spearman correlation. Fatigue score was moderately significantly correlated with sleep quality score and depression score (r=0.505, P=<0.001; and r=0.525, P=<0.001), respectively. On the other hand, the fatigue score was negatively correlated with GLTEQ, but the association was not statistically significant (r=−0.067; P=0.335) (Table 2).

**Table 2:** Clinical characteristics of patients with MS (n=211). F: frequency; MS: multiple sclerosis; RRMS: relapsing-remitting multiple sclerosis; PPMS: primary progressive multiple sclerosis; SPMS: secondary progressive multiple sclerosis; DMT: disease-modifying therapy.

Variable	F (%)
Type of MS	
RRMS	168 (79.6)
PPMS	32 (15.2)
SPMS	11 (5.2)
Taking DMT medications	
Yes	157 (74.4)
No	54 (25.6)
Presence of other chronic illness	
Yes	33 (15.6)
No	178 (84.4)
Use of vitamin D	
Yes	144 (68.2)
No	67 (31.8)
Use of multivitamins	
Yes	127 (60.2)
No	84 (39.8)
Fatigue	
Yes	78 (37)
No	133 (63)
Exercise	
Active	78 (37)
Inactive	133 (63)
Depression	
Yes	114 (54)
No	97 (46)

Table 3 shows the association between sociodemographic variables, clinical variables, and fatigue using the chi-square test and simple logistic regression. For simple logistic regression, reference groups were chosen based on the least fatigue percentage among the groups. It was found that females were 1.5 times more likely to develop fatigue compared to male patients (95% CI: 0.8-2.8; P=0.125).

The odds of having fatigue among married patients compared to unmarried OR=0.9 (95% CI: 0.5-1.7; P=0.874). The OR of fatigue among young age patients was 0.183 (95% CI: 0.956-0.035; P-value: 0.044), and the OR among elderly patients was 0.184 (95%CI: 0.954-0.035; P-value: 0.044). Unemployed was found to have OR: 0.445 (95% CI: 0.24-0.80; P-value: 0.007) compared to students.

According to income, patients with an income of fewer than 4000 Riyals were 4.6 times more likely to develop fatigue compared to those with an income of 8000-11999 Riyals (95%CI: 1.4-15.2; P-value: 0.011), and those with an income of 12,000-15,999 Riyals were 3.3 times more likely to develop fatigue compared to those with an income of 8000-11,999 Riyals (95% CI: 1.1-10.2; P-value: 0.033).

According to weight, underweight MS patients were 80% less likely to develop fatigue compared to overweight patients (95% CI: 0.1-0.7; P-value: 0.008).

The most commonly prevalent type of MS among patients is RRMS; therefore, it was preferred to compare the outcome between common and rare types (PPMS and SPMS) to obtain meaningful evidence.

There was a statistically significant association between progressive types of MS compared to RRMS and the presence of fatigue (OR=4.4; 95% CI: 2.1-8.9; P=0.000), and those diagnosed 10 years ago and more were 2.7 times more likely to develop fatigue compared to those diagnosed seven to nine years ago (95% CI: 1.0-7.3; P-value: 0.049).

A significant association was found between depression and the presence of fatigue (OR=9.740; 95% CI: 4.790-19.803; P=<0.001). The presence of other chronic diseases was significantly associated with fatigue (OR=2.360; 95% CI: 1.1‑5.008, P=0.023). Those who were physically inactive were 1.07 times more likely to develop fatigue compared to active patients (95% CI: 0.6-1.9; P=0.805) (Table 3).

**Table 3:** Association between fatigue and patients’ characteristics (n=211). N: number; MS: multiple sclerosis; RRMS: relapsing-remitting multiple sclerosis; PPMS: primary progressive multiple sclerosis; SPMS: secondary progressive multiple sclerosis; DMT: disease-modifying therapy; OR: odd ratio.

Variable		Presence of fatigue		OR (95% CI)	P-value
		Yes N (%)	No N (%)		
Gender	Female	54 (40.9)	78 (59.1)	1.5 (0.8-2.8)	0.125
	Male	24 (30.4)	55 (69.6)		
Age	Young age	38 (35.5)	69 (64.5)	0.183 (0.053-0.956)	0.044
	Old age	6 (75)	2 (25)	0.184 (0.053-0.954)	0.044
	Middle age	34 (35.4)	62 (64.6)	Reference	
Marital status	Married	49 (36.6)	85 (63.4)	0.9 (0.5-1.7)	0.874
	Unmarried	29 (37.7)	48 (62.3)		
Employment status	Employed	27 (28.7)	67 (71.3)	0.255 (0.068-0.949)	0.042
	Unemployed	48 (47.5)	53 52.5	0.445 (0.246-0.805)	0.007
	Student	3 (18.8)	13 (81.3)	Reference	
Educational level	School	37 (46.8)	42 (53.2)	0.315 (0.067-1.479)	0.143
	Postgraduate studies (reference)	4 (57.1)	3 (42.9)	0.661 (0.139-3.147)	0.603
	University	37 (29.6)	88 (70.4)	Reference	
Income	Less than 4000	14 (53.8)	12 (46.2)	4.6 (1.4-15.2)	0.011
	7999-4000	14 (40)	21 (60)	2.6 (0.8-8.1)	0.086
	15,999-12,000	16 (45.7)	19 (54.3)	3.3 (1.1-10.2)	0.033
	19,999-16,000	15 (32.6)	31 (67.4)	1.9 (0.6-5.7)	0.234
	More than 20,000	13 (33.3)	26 (66.7)	2 (0.6-6.1)	0.223
	11,999-8000	6 (20)	24 (80)	Reference	
BMI categories	Underweight	5 (41.7)	7 (58.3)	0.297 (0.121-0.726)	0.008
	Normal weight	37 (36.6)	64 (63.4)	0.546 (0.141-2.120)	0.382
	Obese	17 (56.7)	13 (43.3)	0.442 (0.193-1.012)	0.053
	Overweight	19 (27.9)	49 (72.1)	Reference	
Living	City	72 (36)	128 (64)	0.4 (0.1-1.5)	0.336
	Village	6 (54.5)	5 (45.5)		
Type of house	Own house	46 (35.9)	82 (64.1)	0.8 (0.5-1.5)	0.700
	Rental house	32 (38.6)	51 (61.4)		
Smoking	Yes	22 (40)	33 (60)	1.1 (0.6-2.2)	0.588
	No	56 (35.9)	100 (64.1)		
Family history of MS	Yes	17 (38.6)	27 (61.4)	1.0 (0.5-2.1)	0.797
	No	61 (36.5)	106 (63.5)		
Type of MS	Progressive types (PPMS and SPMS)	28 (65.1)	15 (34.9)	4.4 (2.1-8.9)	<0.001
	RRMS	50 (29.8)	118 (70.2)		
Time since diagnosis	Less than a year	5 (33.3)	10 (66.7)	1.2 (0.3-4.8)	0.746
	1-3 Years	28 (34.1)	54 (65.9)	1.2 (0.5-3.3)	0.588
	4-6 Years	12 (31.6)	26 (68.4)	1.1 (0.3-3.3)	0.79
	10 Years and more	25 (52.1)	23 (47.9)	2.7 (1.0-7.3)	0.049
	7-9 Years	8 (28.6)	20 (71.4)	Reference	
Taking DMT	Yes	61 (38.9)	96 (61.1)	1.3 (0.7-2.6)	0.333
	No	17 (31.5)	37 (68.5)		
Using vitamin D	Yes	59 (41)	85 (59)	1.7 (0.9-3.2)	0.077
	No	19 (28.4)	48 (71.6)		
Use of multivitamins	Yes	48 (37.8)	79 (62.2)	1.0 (0.6-1.9)	0.759
	No	30 (35.7)	54 (64.3)		
Depression	Yes	66 (57.9)	48 (42.1)	9.7 (4.7-19.8)	<0.001
	No	12 (12.4)	85 (87.6)		
Sleeping hour	Before midnight	16 (27.1)	43 (72.9)	0.5 (0.2-1.0)	0.065
	After midnight	62 (40.8)	90 (59.2)		
Sleep quality	Poor	65 (48.5)	69 (51.5)	4.6 (2.3-9.2)	<0.001
	Good	13 (16.9)	64 (83.1)		
Presence of another chronic disease	Yes	18 (54.5)	15 (45.5)	2.3 (1.1-5.0)	0.023
	No	60 (33.7)	118 (66.3)		
Exercise	Inactive	50 (37.6)	83 (62.4)	1.07 (0.6-1.9)	0.805
	Active	28 (35.9)	50 (64.1)		

Table 4 shows the results of multiple logistic regression. Among BMI categories, obese patients were 8.3 times more likely to develop fatigue compared to overweight (95% CI: 2.4-28.7; P-value: 0.001).

The results show that unemployed patients were 2.8 times more likely to develop fatigue compared to students (95% CI: 1.1-45.0; P-value: 0.032). Patients with income less than 4000 Riyals and 4000-7999 Riyals were 7.1 times and six times more likely to develop fatigue compared to the reference group (95% CI: 1.5-33.5 and 1.3-27; P-value: 0.013 and 0.019).

We found that the OR of fatigue among progressive types was 9.4 (95%CI: 3.3-26.6; P-value: <0.001) compared to the relapsing-remitting type. Those who had the progressive type were nine times more likely to have fatigue compared to the relapsing-remitting type (95%CI: 3.3-26.6; P-value: <0.001).

According to sleep quality, those with poor sleep quality were 5.6 times more likely to develop fatigue compared to those with good sleep quality (95% CI: 2.2-14.4; P-value: <0.001). Depressed patients were 12.3 times more likely to have fatigue compared to non-depressed patients (95% CI: 5-30.4; P-value: <0.001) (Table 4).

This model can predict 56% of fatigue among MS patients (R^2^: 0.56) (Table 4).

**Table 4:** Multiple logistic regression model. MS: multiple sclerosis; RRMS: relapsing-remitting multiple sclerosis; PPMS: primary progressive multiple sclerosis; SPMS: secondary progressive multiple sclerosis; BMI: body mass index; OR: odd ratio.

Variable		OR	95% CI	P-value
BMI	Underweight	6.8	0.7-62.0	0.087
	Normal weight	2.7	1.0-7.0	0.043
	Obesity	8.3	2.4-28.7	0.001
	Overweight	Reference		
Employment	Employed	2.8	0.4-17.6	0.259
	Un-employed	7.3	1.1-45.0	0.032
	Student	Reference		
Type of MS	Progressive types (PPMS and SPMS)	9.4	3.3-26.6	<0.001
	Relapsing remitting	Reference		
Income	Less than 4000 Riyals	7.1	1.5-33.5	0.013
	4000-7999 Riyals	6.0	1.3-27.0	0.019
	15,999-12,000 Riyals	3.6	0.8-15.5	0.085
	19,999-16,000 Riyals	2.8	0.6-12.5	0.155
	More than 20,000 Riyals	2.6	0.5-11.8	0.204
	Riyals 11,999-8000	Reference		
Sleep quality	Poor	5.6	2.2-14.4	<0.001
	Good	Reference		
Depression	Yes	12.3	5.0-30.4	<0.001
	No	Reference		

## Discussion

The incidence of fatigue among MS reached 90% in some studies. The most common MS symptom patients describe is fatigue. Fatigue deeply affects the social, professional, and familial domains of patients with MS. This results in significant health costs, and hence, fatigue should not be neglected. It is necessary to understand this symptom and adopt novel therapeutic approaches to manage it. Hence, the current study aimed to investigate the role of exercise on fatigue among patients with MS [16,17].

Fatigue prevalence among patients with MS in the present study was significant (37%). But previous studies have reported a higher prevalence of MS fatigue. A study in India by Nagaraj et al. documented that 58.1% of patients with MS had fatigue [18]. Lerdal et al. assessed the population with MS in Oslo and found the prevalence of fatigue was 60% [19]. Another study by Rooney et al. conducted in Australia, the United States (US), and the United Kingdom (UK) through national and international MS charities and organizations in 498 patients with MS found the prevalence of fatigue was 69% [20]. NARCOMS also found that the prevalence of severe fatigue among patients with MS was 74% [8]. In Norway, Broch et al. found that among 1182 patients with MS, 81% had fatigue [21]. This variation in fatigue prevalence might be interpreted through the differences in the way study subjects are chosen (since in the present study, patients with severe psychiatric disorders, other comorbidities that interfered with physical activities, and MS patients with terminal illness and pregnancy were excluded), methods used to assess fatigue, study time, and the size of the study population. In our study, we used MFIS to measure fatigue among 211 patients over four months; 50.7% of responders were young, and obesity was 14.2%. Besides these factors, the small sample size might be the cause of the lower prevalence of fatigue in our study compared to other studies.

Generally, fatigue can occur at all stages of MS. MS-related fatigue is distinguished into two types: primary fatigue, which is related to disease-specific mechanisms. Secondary fatigue is related to MS complications (reduced activity, sleep disorders, depression, and treatment adverse events) [17,22]. There are efforts to recognize the factors that contribute to fatigue among patients with MS [18].

Lately, nonpharmacological methods have been used to treat fatigue in patients with MS, such as physical exercise (aerobic work), cognitive behavioral therapy, cryostimulation, and energy conservation strategies [23]. Routine physical activity is highly recommended in MS fatigue cases as accumulated data has shown [24,25]. Patients with MS who are not physically active can be exposed to complications such as heart problems, osteoporosis, obesity, and deconditioning, while regular exercise might play a significant role in neuroprotection and help with spasticity and muscle strengthening [16]. More than 50% of patients in the current study did not exercise, which reveals the need to encourage physical activity and exercise among these patients. However, it should be considered that fatigue increases a higher level of disability, which in turn may lead to reduced motivation for exercise or physical activity [26]. Alsaedi et al. mentioned that younger age has a lower Expanded Disability Status Scale (EDSS), also time of diagnosis at a younger age, lower BMI, RRMS, and a duration of fewer than five years associated with a lower EDSS [27]. This, in turn, indicates that there is a need to identify factors for not exercising among patients with MS and try to find solutions to stimulate physical activity and exercise in those patients. In our study, 63% of the responders among all age groups were physically inactive (among young patients, 64% were physically inactive while only 35.5% were physically active). There was no correlation between exercise and fatigue in this study. A review suggested that the studies that evaluated the role of exercise treatment on MS fatigue showed various results. The general interpretation, though, is that exercise therapy has the potential to reduce fatigue in patients with MS [28]. The majority of patients with MS in the present study were female, which is compatible with the findings of many previous studies [29-33].

According to the current results, there is no association between fatigue and gender. This finding is not compatible with that of Broch et al., who suggested that fatigue was more prevalent in women [21]. Generally, men and women have different responses to pain due to genetics and psychosocial factors. This can lead to differences in tolerance to pain, sensitivity to it, and reporting it [34]. 

The current study showed that the patient's BMI is one of the factors predicting fatigue among patients. These findings are consistent with Rezaeimanesh et al.'s study, which found a positive correlation between BMI and fatigue score among 85 MS patients (P-value: 0.031; r: 0.23). Such findings highlight the importance of carrying out a comprehensive approach when dealing with MS patients, including weight management alongside pharmacotherapy treatment [35].

Recently, sleep disorders and their potential role in fatigue among patients with MS have been gaining attention. Specific sleep problems or disorders that may disproportionately affect patients with MS include restless legs syndrome (RLS), periodic limb movement disorder, circadian rhythm disturbances, and chronic insomnia [36]. The relationship between sleep disorders and fatigue in MS is still controversial, and the causes of and relationship between sleep disorders and fatigue in MS are not yet fully understood [37]. In the current study, the majority of patients with MS (64%) had poor sleep quality. The current findings are compatible with that of Nociti et al., who found a strict relation between fatigue and sleep disorders, in which MS was linked to a high prevalence of sleep complaints, including restless legs syndrome (RLS), excessive daytime sleepiness, subjectively poor sleep quality, and symptoms of obstructive sleep apnea syndrome (OSAS) [37]. The current study also showed a significant association between sleep quality and fatigue. Ghaem and Haghighi [38] also found that 87.2% of patients with MS in their study suffered from poor sleep.

The QOL of a patient is impacted by MS in part due to physical disability [39]. Depression also commonly accompanies MS with a prevalence of about 50% [36]. In the current study, the prevalence of depression among patients with MS was around 54%, and there was a significant association between depression and the presence of fatigue. These findings are compatible with those of previous studies [18,20,39].

Depression itself can be obvious from fatigue because the symptoms of fatigue are often mistaken, which makes this condition difficult to distinguish from MS fatigue. Depression should be addressed and treated regardless of any contribution to fatigue because it has a fundamental impact on QOL. MS severity and its subtype can influence the risk of fatigue. Patients with progressive subtypes of MS experience severe fatigue [36]. In the present study, there was a protective association between RRMS and fatigue compared to other types (PPMS and SPMS). This protective association might be explained by the nature of RRMS compared to the more complex aggressive types (PPMS and SPMS). Broch et al. have shown inconsistent results, as there was no significant difference in the rate of fatigue among patients with progressive MS and those with relapsing MS [21].

The current study has some limitations. Firstly, it was a single central study, and all cases were interviewed within four months, which is how the accuracy and generalizability of the results are affected to some extent. Secondly, data was collected through telephone calls, and disability scores were not collected. Other limitations can be regarding study design and small sample size, recall bias, and limitations in resources. 

## Conclusions

The present study showed that fatigue is a common problem among MS patients, and the majority of patients do not practice exercises, which means there is a need to encourage physical activity and exercise among the patients, in addition, to applying for a regular exercise program as a part of their rehabilitation program. The current study showed that there is an association between fatigue and progressive MS types, low income, unemployment, obesity, depression, and poor sleep quality, but the association between exercise and the resolution of fatigue symptoms was not significant. Further studies on the same issue are needed with the importance of comprehensive care and holding psychological support meetings, sleep counseling to improve sleep quality and proper weight management plans for the patients.
